# Deworming Coverage and Associated Factors Among Children Aged 12–59 Months in Sub‐Saharan Africa: A Multilevel Poisson Regression Analysis Using Demographic and Health Survey Data, 2021–2024

**DOI:** 10.1155/jotm/7289470

**Published:** 2026-09-22

**Authors:** Temesgen Gebeyehu Wondmeneh

**Affiliations:** ^1^ Department of Public Health, College of Medical and Health Science, Samara University, Semera, Ethiopia, su.edu.et

**Keywords:** children aged 12–59 months, Demographic and Health Survey data, deworming coverage, multilevel Poisson regression, sub-Saharan Africa

## Abstract

**Background:**

Under the revised Demographic and Health Survey‐8 (DHS‐8) guidelines, deworming coverage is assessed only among children aged 12–59 months in accordance with World Health Organization recommendations. However, evidence for this age group in sub‐Saharan Africa remains limited.

**Objective:**

To assess deworming coverage and associated factors among children aged 12–59 months in sub‐Saharan Africa.

**Methods:**

A cross‐sectional study was conducted using DHS data collected between 2021 and 2024 from 11 sub‐Saharan African countries. A weighted sample of 96,830 children aged 12–59 months was included. Multilevel Poisson regression with a log link and robust variance estimation was used to estimate adjusted prevalence ratios (APRs) and 95% confidence intervals (CIs).

**Results:**

Overall, 47,426 of 96,830 children received deworming medication, giving coverage of 48.98% (95% CI: 48.67–49.30). Deworming coverage ranged from 32.0% in Burkina Faso to 66.0% in Kenya. Higher deworming prevalence was observed among children of mothers aged 25–34 years (APR = 1.04, 95% CI: 1.02–1.06) and 35–49 years (APR = 1.08, 95% CI: 1.06–1.10), those whose mothers had primary (APR = 1.19, 95% CI: 1.16–1.22), secondary (APR = 1.20, 95% CI: 1.18–1.26), or higher education (APR = 1.29, 95% CI: 1.20–1.34), and those who received vitamin A supplementation (APR = 2.75, 95% CI: 2.67–2.83). Children aged 36–59 months were also more likely to receive deworming medication (APR = 1.09, 95% CI: 1.08–1.11). Lower deworming prevalence was observed among children from households with ≥ 5 members (APR = 0.97, 95% CI: 0.95–0.98) and among those whose mothers reported distance to health facilities as a major problem (APR = 0.94, 95% CI: 0.92–0.96).

**Conclusion:**

Nearly half of eligible children received deworming medication in sub‐Saharan Africa. Strengthening integrated maternal and child health services, improving women’s education and decision‐making capacity, and expanding access to services in underserved communities are crucial to increase deworming coverage.

## 1. Introduction

Soil‐transmitted helminth (STH) infections remain a major public health concern, particularly in sub‐Saharan Africa, where they contribute to malnutrition, anemia, impaired growth, and cognitive deficits among children under 5 years of age [1–3]. Deworming interventions using preventive chemotherapy are recognized as an effective strategy to reduce the burden of these infections and improve child health outcomes [4]. Accordingly, the World Health Organization (WHO) recommends periodic deworming for children in endemic areas, with coverage and adherence serving as key indicators of program success [5]. Treatment is advised once yearly when community prevalence exceeds 20% and twice yearly when it exceeds 50% to reduce morbidity by lowering worm burden [6]. Despite these global efforts, deworming coverage remains suboptimal in many countries [7], emphasizing the need for robust data to inform targeted interventions.

Deworming coverage among preschool‐aged children varies substantially across countries and regions. In low‐ and middle‐income countries, 33.0% of mothers reported that their child had received deworming medication during the 6 months preceding the survey, with estimates ranging from 5.1% in Azerbaijan to 73.6% in Rwanda [8]. In sub‐Saharan Africa, a pooled analysis of Demographic and Health Survey (DHS) data collected before 2020 reported a deworming coverage of 45.0% among children under five years of age, with country‐specific estimates ranging from 41.8% in Malawi to 50.5% in Lesotho [9]. Another study conducted in East Africa found a deworming coverage of 54.1% among children aged 12–59 months [10]. In Ethiopia, deworming coverage among children aged 12–59 months ranged from 13.3% to 15.1% [11, 12].

To strengthen control efforts, partners have formed alliances to expand deworming programs, integrate water, sanitation, and hygiene (WASH) initiatives, and support research and monitoring of STH infections in children [13]. The United Nations International Children’s Emergency Fund (UNICEF) reports that deworming tablets are provided to children aged 24–59 months through integrated campaigns alongside measles vaccination and vitamin A supplementation, highlighting their role in improving the effectiveness of broader child health interventions. For example, in Ethiopia, these campaigns reached approximately 75%–80% of eligible children aged 12–59 months who received a dose of deworming medicine [14, 15].

Uptake of deworming is influenced by maternal and child characteristics. Mothers who were educated, older, employed, wealthier, attended antenatal care (ANC), delivered in health facilities, exposed to media, and made their own decisions, as well as children who had diarrhea or received vitamin A supplementation, were more likely to be dewormed. Conversely, mothers with many children were less likely to deworm their children [9–12].

Most previous studies on deworming coverage have focused on children aged 6–59 months. However, the revised DHS guideline introduced after 2020 redefined the deworming indicator to include only children aged 12–59 months [16]. Because children aged 6–11 months are not eligible for deworming under WHO recommendations, their exclusion is expected to produce more accurate estimates that better reflect program performance. Consequently, earlier studies that included children aged 6–59 months may have obscured important age‐specific differences. Furthermore, factors influencing deworming uptake among children aged 6–11 months likely differ from those affecting the eligible 12–59‐month group. Despite this revision, evidence on deworming coverage among children aged 12–59 months following the 2020 DHS guideline remains limited. In addition, the application of multilevel Poisson regression with a log link and robust variance allows for the direct estimation of prevalence ratios, provides valid inference even when Poisson assumptions are partially violated, and ensures reliable convergence in clustered datasets. Therefore, the aim of this study was to assess the coverage of deworming and its associated factors among children aged 12–59 months in sub‐Saharan Africa after 2020.

## 2. Methods

### 2.1. Study Design and Setting

This study employed a cross‐sectional design using the most recent standard DHS data collected between 2021 and 2024 from 11 eligible sub‐Saharan African countries. These countries were included because they had publicly available DHS‐8 standard survey data containing the revised deworming indicator for children aged 12–59 months at the time of data extraction. The DHS surveys are nationally representative household surveys that use standardized questionnaires and data collection procedures across participating countries.

### 2.2. Data Source

This study used publicly accessible data from the DHS Program, specifically the most recent standard DHS conducted between 2021 and 2024 in the 11 eligible sub‐Saharan African countries. Access to the datasets was obtained by registering as a DHS user and selecting the required survey samples and recoding files through the DHS website (https://dhsprogram.com/data/).

Reporting guideline: This study was reported in accordance with the Strengthening the Reporting of Observational Studies in Epidemiology (STROBE) guideline for cross‐sectional studies (Supporting information 1).

### 2.3. Eligibility Criteria, Study Population, and Sample Size

The study population comprised living children aged 12–59 months who were eligible to receive deworming medication within the six months preceding the survey, based on the most recent DHS data (2021–2024) from 11 eligible sub‐Saharan African countries. Only living children with a recorded deworming status (yes/no) were included. Children younger than 12 months, those who had died before the survey, and those with missing or unknown deworming status were excluded. After applying these criteria, the final analytic unweighted sample comprised 100,999 children aged 12–59 months from the 11 sub‐Saharan African countries included in the study (Table 1).

**TABLE 1:** Number of participants by country.

Country	Survey years	Sample size	Percentages (%)
Tanzania	2022	8044	8.0
Senegal	2023	7868	7.8
Mozambique	2022/23	6751	6.7
Lesotho	2023/24	1780	1.7
Kenya	2022	14,711	14.6
Ghana	2022	7076	7.0
DRC	2020/24	17,236	17.1
Burkina Faso	2021	9272	9.2
Côte d’Ivoire	2021	7688	7.6
Mali	2023/24	11,474	11.3
Madagascar	2021	9099	9.0
Total		100,999	100

### 2.4. Sampling Procedures

**FIGURE 1:**
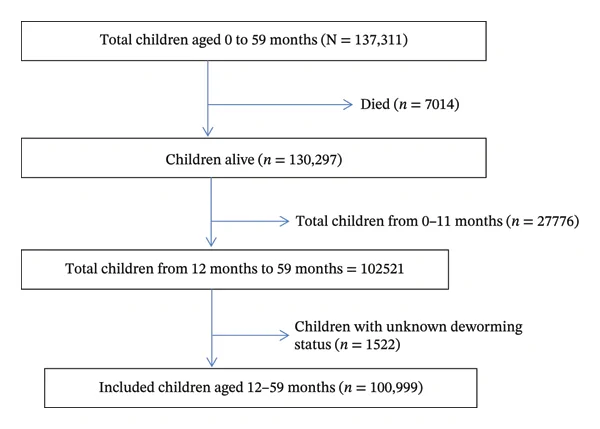
Selection procedure for children aged 12–59 months.

The study used the most recent DHS data (2021–2024) from 11 eligible sub‐Saharan African countries. DHS employs a multistage, stratified cluster sampling design**,** with enumeration areas (EAs) or clusters as primary sampling units (PSUs) selected with a probability proportional to the size within strata, typically defined by region and urban/rural residence. Within each cluster, households are systematically sampled, and eligible individuals are enumerated and interviewed [16]. The initial datasets from 11 countries included 137,311 children aged 0–59 months. Of these, 7014 children had died, leaving 130,297 living children. Children younger than 12 months (*n* = 27,776) were excluded from the analysis, resulting in 102,521 children aged 12–59 months. Furthermore, 1522 children with unknown deworming status were excluded, yielding a final analytic unweighted sample of 100,999 children aged 12–59 months (Figure 1).

### 2.5. Variables

#### 2.5.1. Dependent Variable

The dependent variable was the child’s deworming status, obtained from the DHS dataset (Kids Recode [KR] file) using variable h43. This variable indicates whether a living child aged 12–59 months received deworming medication within the six months preceding the survey interview. It was coded as 1 = Yes (the child received deworming medication) and 0 = No (the child did not receive deworming medication).

#### 2.5.2. Independent Variables

The independent variables included in this study were selected based on previous literature [9, 10] and their availability in the dataset. Individual‐level variables such as the mother’s education, the sex of the household head, the sex of the child, vitamin A supplementation, and diarrhea were obtained directly from the DHS KR file without recoding. Based on DHS data, mothers’ education was categorized into no education, primary, secondary, and higher. The mother’s age was grouped into three categories by combining adjacent age groups: 15–24 years (15–19 and 20–24), 25–34 years (25–29 and 30–34), and 35–49 years (35–39, 40–44, and 45–49). Child age was categorized into two groups: 12–35 months and 36–59 months. The wealth index was classified into three categories by combining the original wealth groups as follows: poor (poorest and poorer), middle, and rich (richer and richest). Women were coded as having **decision-making power (Yes = 1)** if they usually made their own health care decisions, whereas decisions made jointly with a partner, by the partner alone, or by someone else were coded as (**No = 0)**, indicating no independent decision‐making. Similarly, individuals were coded as **literate (Yes = 1)** if they attended school beyond the secondary level or could read part or all of a sentence, while those who had no formal education or attended school only up to the secondary level and could not read at all were coded as **illiterate (No = 0)**. An individual was considered to have media exposure if they were exposed to at least one form of media such as listening to the radio, watching television, or reading newspapers at least once a week, whereas those who were not exposed to any of these media at least once a week were classified as having no media exposure. Household size was recoded into two groups: households with 1–4 members and those with 5 or more members. Marital status was grouped into two categories: **married** for those living with their husband or partner and **unmarried** for those who were divorced, widowed, separated, not living together, or never married. Birth order is coded as the **first birth** if it is the mother’s first child; all other births are coded as **subsequent births.** ANC visits were grouped as no visits, 1–2 visits, 3 visits, and 4 or more visits. Children under five who had diarrhea in the 2 weeks preceding the survey and for whom advice or treatment was sought were coded as 1, and those for whom no advice or treatment was sought were coded as 0. Children under five who received a vitamin A supplement were coded as Yes (1), and those who did not were coded as No (0). Community‐level factors such as place of residence (urban or rural) and perceived distance to health facilities (a big problem or not) were taken directly without aggregating individual data. However, community literacy and media exposure were categorized as “high” or “low” based on the median values within each cluster, while the community wealth index was similarly derived from individual household wealth levels and classified according to the cluster median.

### 2.6. Outcome Measurement

According to DHS‐8 guidelines, a child is considered to have received deworming medication if they were given it in the 6 months before the survey; otherwise, they are considered not to have received it. This is calculated as the proportion of living children aged 12–59 months who received deworming medication out of the total number of children in the same age group.

Child deworming status (%) = (number of children aged 12–59 months who received deworming in the past 6 months/total number of living children aged 12–59 months) × 100 [16].

### 2.7. Operational Definitions Based on DHS‐8 Guidelines [16]

**Improved water sources** include piped water into the dwelling, yard, or plot; public taps or standpipes; piped water to a neighbor’s residence; tube wells/boreholes; protected wells or springs; rainwater; tanker trucks; carts with small tanks; and bottled water.

**Unimproved water sources** include unprotected wells, unprotected springs, surface water (from rivers, dams, lakes, ponds, streams, canals, or irrigation channels), and other unspecified sources.

**Improved sanitation facilities** include flush toilets connected to a piped sewer system, septic tank, or pit latrine; ventilated improved pit latrines; pit latrines with a slab; composting toilets; and flush toilets with an unknown destination.

**Unimproved sanitation facilities** include flush toilets that discharge elsewhere, pit latrines without a slab or with open pits, bucket toilets, hanging toilets or latrines, and other unspecified facilities.

### 2.8. Data Collection Methods, Tools, and Quality Control

The data for this study were collected through the DHS Program, which conducts standardized, nationally representative household surveys. Data collection followed a multistage process, using structured questionnaires administered to women with reproductive age (15–49 years) and, where relevant, their children under five. The questionnaires covered household characteristics, maternal and child health, nutrition, and child deworming practices. Data were collected by trained field enumerators under the supervision of field supervisors**.** Pretesting and pilot surveys ensured clarity, cultural relevance, and consistency of the tools. Data were collected using standardized electronic questionnaires administered by trained fieldworkers and entered into secure DHS databases using standardized coding procedures. Quality control measures included supervisory spot checks and centralized data cleaning. Consistency checks and validation rules were applied during and after data entry to detect discrepancies. Additionally, weighting, stratification, and clustering adjustments were incorporated in the analysis to account for the complex survey design and ensure reliable, nationally representative estimates [16].

### 2.9. Data Management and Statistical Analysis

The data were extracted and analyzed from the KR dataset of DHS using Stata Version 17.0 statistical software (StataCorp LLC, College Station, TX, USA). The DHS datasets are publicly available and fully de‐identified, ensuring the confidentiality of all participants. Prior to analysis, the data were weighted using the sampling weight variable (v005) to adjust for unequal probabilities of selection and nonresponse. Applying these weights ensures that the estimates are nationally representative and unbiased across different regions and population groups. Frequency and percentage were used to describe categorical variables. Missing data were assessed using the Stata commands misstable summarize and misstable pattern, which generated summaries of variable‐level completeness and overall patterns of missingness, respectively. Missing values were identified in five variables. Diarrhea and vitamin A supplementation had less than 1% missing data and were considered essentially complete, with no imputation performed. In contrast, distance to a health facility, women’s decision‐making power, and ANC visits showed moderate to substantial missingness. ANC visits exhibited the highest proportion of missing values (56.0%). This substantial missingness primarily reflects the DHS survey structure, in which ANC information is collected only for the most recent birth within a specified reference period and is therefore unavailable for many children included in the pooled analysis. Because ANC utilization is a well‐established determinant of child preventive health service uptake and was considered theoretically important for explaining deworming practices, excluding this variable could have introduced omitted‐variable bias and substantially reduced the analytical sample. Therefore, ANC visits were retained and missing values were addressed using multiple imputation by chained equations (MICE). ANC visits, an ordinal variable, were imputed using ordinal logistic regression, whereas women’s decision‐making power and distance to a health facility, which were binary variables, were imputed using logistic regression models. The imputation models included child deworming status, place of residence, and maternal age as predictor variables. Twenty imputed datasets were generated with a burn‐in period of 10 iterations and 200 total iterations to ensure convergence and stability. The resulting datasets were combined according to Rubin’s rules, and all subsequent analyses were performed using the imputed data. To evaluate the robustness of the findings, a sensitivity analysis was performed by comparing the final multilevel Poisson regression model fitted to the multiply imputed dataset with the corresponding complete‐case analysis. The direction, magnitude, and statistical significance of adjusted prevalence ratios (APRs) obtained from both analyses were compared to assess the influence of missing data handling on the study conclusions. The DHS employs a multistage stratified cluster sampling design, resulting in a hierarchical data structure in which children are nested within households and households within clusters. This clustering introduces intraclass correlation, whereby observations within the same cluster are more similar than those from different clusters. Given the hierarchical structure of the data and the binary outcome (child deworming status), a multilevel generalized linear mixed model with a Poisson distribution, log link function, and robust variance estimation was fitted to estimate APRs while accounting for within‐cluster correlation. Because the prevalence of child deworming exceeded the threshold at which odds ratios may overestimate associations [17], Poisson regression with a log link was used to directly estimate prevalence ratios [18]. Although originally developed for count data, this approach is widely recommended for estimating prevalence ratios for common binary outcomes. Random intercepts were included to account for cluster‐level variation, while robust variance estimation provided valid standard errors and confidence intervals (CIs) even when the Poisson variance assumption was violated. Variables with a *p* value < 0.20 in the bivariable multilevel Poisson regression analysis were included in the multivariable multilevel Poisson regression model. All statistical tests were two‐sided, and statistical significance was declared at *p* < 0.05. APRs with 95% CIs described the magnitude and precision of associations. Before conducting multilevel (mixed‐effects) Poisson regression, multicollinearity among independent variables was assessed using the variance inflation factor (VIF) in Stata. Values above 5 indicate potential multicollinearity, while values below 5 are generally considered acceptable. All variables included in the analysis were checked for multicollinearity. A multilevel (mixed‐effects) Poisson regression model (log link with robust variance estimation) was developed in four stages. The null model (Model I), which included no predictors, assessed between‐cluster variation in child deworming uptake and justified multilevel modeling by estimating the intraclass correlation coefficient (ICC) and cluster‐level variance. Model II included only individual‐level variables to evaluate their associations with the outcome while accounting for clustering. Model III included only community‐level variables to examine their contribution to between‐cluster variation. Finally, Model IV (full model) incorporated both individual‐ and community‐level variables to estimate adjusted associations and quantify the proportion of variance explained at each level using the proportional change in variance (PCV). Model fitness was evaluated using several statistical criteria to ensure the adequacy and goodness of fit of the multilevel Poisson regression models. The −2 log likelihood (−2LL) and Akaike information criterion (AIC) were used to compare successive models, with lower values indicating improved model fit. The likelihood ratio test was also applied to assess the significance of random effects by comparing the multilevel model to a standard (single‐level) Poisson model. A significant likelihood ratio test (*p* < 0.05) confirmed the presence of meaningful cluster‐level variation. Additionally, PCV was used to quantify the reduction in between‐cluster variance after the inclusion of explanatory variables, reflecting improved model performance. To quantify cluster‐level variation in the multilevel Poisson regression model (log link with robust variance estimation), three measures of heterogeneity were computed: ICC, median rate ratio (MRR), and PCV. The ICC represents the proportion of total variance attributable to between‐cluster differences and was calculated using the formula $\text{ICC}=\sigma_{u-\text{null}}^{2}/\left(\pi^{2}/\sigma_{u-\text{null}}^{2}\right)$, where $\sigma_{u}^{2}$ denotes the cluster‐level variance (random intercept variance). MRR quantifies the median difference in the outcome between individuals from clusters with higher and lower risks, randomly selected from the population, and was estimated as $\text{MRR}=\exp\left(0.6745\times \sqrt{\left(2\sigma_{u-\text{model}}^{2}\right)}\right)$. MRR value of 1 indicates no cluster‐level heterogeneity, while higher values signify greater between‐cluster variation. The PCV measures the proportion of cluster‐level variance explained by the addition of variables to the model, showing how much heterogeneity is reduced compared to the null model. PCV was calculated as = $\left(\left(\sigma_{u-\text{null}}^{2}-\sigma_{u-\text{model}}^{2}\right)/\sigma_{u-\text{null}}^{2}\right)\times 100$, where $\sigma_{u-\text{null}}^{2}$ is the variance from the empty (null) model and $\sigma_{u-\text{model}}^{2}$ is the variance from the adjusted model.

### 2.10. Ethical Approval

Permission to use the data for this analysis was obtained from the DHS Program. Access to the de‐identified datasets was granted following a formal data request through the DHS website (https://dhsprogram.com/data/Access-Instructions.cfm). The datasets are fully anonymized, and no individual participants can be identified. This study strictly followed DHS data use policies and international ethical standards for secondary data research. The DHS Program obtained informed consent from all participants during data collection.

## 3. Results

### 3.1. Description of the Study Participants

A total of 100,999 women with children aged 12–59 months were included in the unweighted sample. After applying the sampling weights, the weighted sample comprised 96,830 participants. Nearly half of the women (46.9%) were aged 25–34 years, and the majorities (86.9%) were married. About half of the children (49.5%) were aged 12–35 months, and 50.5% were males. About 35.5% had no formal education, and over three‐fifths (61.2%) were illiterate. Most households (75.0%) had five or more members, and 79.4% were headed by males. Slightly more than half (54.6%) of the women made their own health‐related decisions. About 44.0% belonged to the poor wealth category, and two‐thirds (66.4%) had media exposure. Similarly, nearly two‐thirds (62.9%) of households had an improved water source, and 50.4% had improved sanitation facilities. Most children (76.2%) were of higher birth order. Nearly two‐thirds (63.3%) of mothers attended four or more ANC visits, while 7.9% had none. About half of the children (50.6%) received vitamin A supplements, and only 12.9% had diarrhea in the 2 weeks preceding the survey. At the community level, 52.6% of communities had high literacy, 55.2% had high media exposure, and 53.3% were classified as high wealth. Most participants (68.6%) lived in rural areas, and 64.1% reported that distance to a health facility was not a major problem (Table 2).

**TABLE 2:** Study participant characteristics.

Variables	Categories	Weighted frequency	Percentage (%)
*Individual levels*			
Women’s age (in year)	15–24	26,086	26.94
	25–34	45,362	46.85
	35–49	25,382	26.21

Marital status	Married	84,129	86.88
	Unmarried	12,701	13.12

Children’s age (in months)	12–35	47,956	49.53
	36–59	48,874	50.47

Children’s sex	Males	48,946	50.55
	Females	47,884	49.45

Women’s education	No education	34,386	35.51
	Primary	29,311	30.27
	Secondary	27,981	28.90
	Higher	5152	5.32

Women’s literacy status	Literate	37,620	38.85
	Illiterate	59,210	61.15

Household size	1–4	24,224	25.02
	≥ 5	72,606	74.98

Household head	Males	76,852	79.37
	Females	19,978	20.63

Women’s decision‐making on health	Jointly/others	38,238	45.45
	Own	45,887	54.55

Wealth index	Poor	42,614	44.01
	Middle	18,937	19.56
	Rich	35,279	36.43

Media exposure	No	32,510	33.57
	Yes	64,320	66.43

Water source	Improved	60,894	62.89
	Unimproved	35,936	37.11

Sanitation facility	Improved	48,797	50.39
	Unimproved	48,033	49.61

Birth order	First	23,089	23.84
	Subsequent	73,741	76.16

ANC visits	No ANC	3372	7.85
	1–2 visits	4637	10.80
	3 visits	7738	18.02
	4 and above	27,202	63.34

Vitamin A supplementation	No	47,475	49.36
	Yes	48,698	50.64

Presence of diarrhea	No	83,945	87.13
	Yes	12,397	12.87

*Community-level factors*			
Community literacy	Low	45,905	47.41
	High	50,925	52.59

Community media exposure	Low	43,357	44.78
	High	53,473	55.22

Community wealth index	Low	45,201	46.68
	High	51,629	53.32

Residence	Urban	30,418	31.41
	Rural	66,412	68.59

Distance to health facility	Not a big problem	58,040	64.13
	Big problem	32,462	35.87

### 3.2. Deworming Status of Children Aged 12–59 Months

**FIGURE 2:**
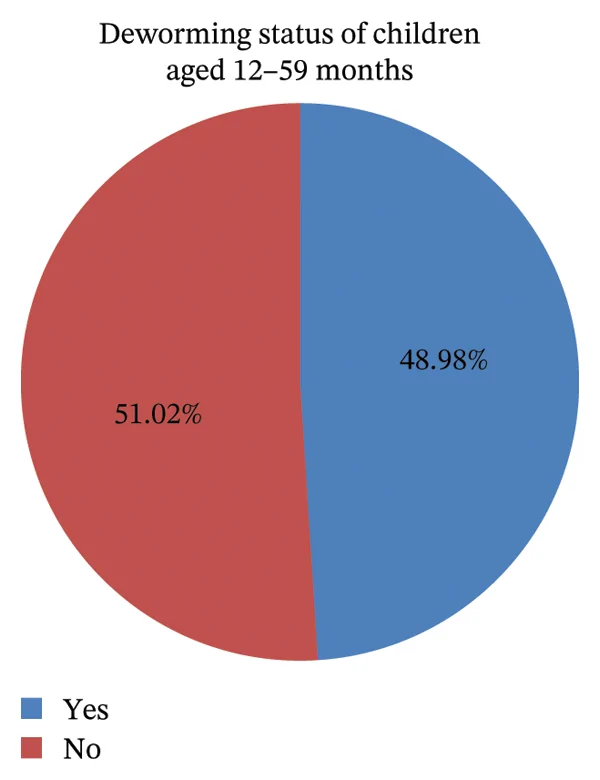
Weighted pooled deworming coverage among children aged 12–59 months in 11 sub‐Saharan African countries.

Among the weighted sample of children, the overall deworming coverage among children aged 12–59 months in sub‐Saharan Africa was 48.98% (95% CI: 48.67–49.30) (Figure 2).

### 3.3. Deworming Coverage of Children Aged 12–59 Months Across Sub‐Saharan African Countries

**FIGURE 3:**
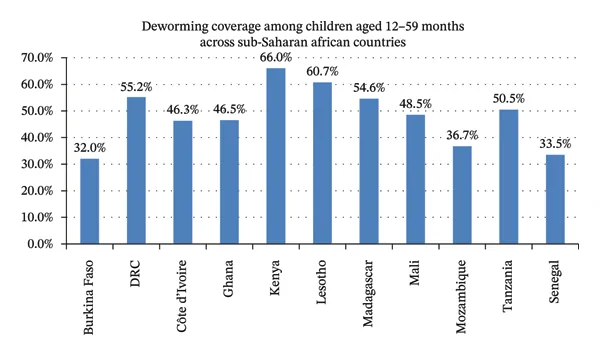
Weighted deworming coverage among children aged 12–59 months by country in 11 sub‐Saharan African countries.

Notable inter‐country disparities in deworming coverage among children aged 12–59 months were observed across sub‐Saharan Africa, ranging from 32.0% in Burkina Faso to 66.0% in Kenya. High coverage was recorded in Kenya (66.0%), Lesotho (60.7%), DRC (55.2%), and Madagascar (54.6%), while moderate levels were seen in Tanzania (50.5%), Mali (48.5%), Ghana (46.5%), and Côte d’Ivoire (46.3%). The lowest coverage occurred in Mozambique (36.7%), Senegal (33.5%), and Burkina Faso (32.0%) (Figure 3).

### 3.4. Missing Data Patterns and Imputation

Based on the missing data patterns, ANC was the primary source of missingness, with 56% of observations incomplete and often missing alone. Despite this high level of missingness, ANC was retained in the analysis because of its theoretical and programmatic importance for child deworming, and its missing values were addressed through multiple imputation to preserve the analytical sample. Decision‐making power (12.5% missing) and distance to the health facility (6.9% missing) also exhibited moderate missingness, occasionally occurring with ANC, and were recommended for imputation to maintain sample size and ensure unbiased estimates. In contrast, vitamin A supplementation (0.6% missing) and diarrhea (0.5% missing) had minimal missingness, which is unlikely to meaningfully affect results, and therefore do not require imputation. Accordingly, MICE was performed to handle missing data for three variables: ANC, distance to the health facility, and women’s decision‐making power. ANC, an ordinal variable, was imputed using ordinary logistic regression, while the other two binary variables were imputed using logistic regression. To ensure accurate and reliable imputations while managing computational constraints and reflecting theoretical relevance to the imputed variables, three fully observed variables, namely, child’s deworming status, place of residence, and maternal age, were used as predictors in all imputation models. A total of 20 imputations were generated with a burn‐in of 10 and 200 iterations to ensure convergence and stability. All missing observations were successfully imputed across the 20 datasets, resulting in a complete analytical sample of 100,999 observations (Supporting Table 1).

### 3.5. Sensitivity Analysis

A sensitivity analysis was conducted by comparing the final multivariable multilevel Poisson regression model using the complete‐case and multiply imputed datasets. Overall, the two analyses produced highly consistent results in terms of the direction, magnitude, and statistical significance of most APRs, indicating that multiple imputation did not materially alter the study findings. Minor differences were observed for women aged 25–34 years, household size, and community media exposure, which reached statistical significance only in the multiple imputation analysis, likely reflecting the greater precision achieved by retaining all available observations. Because multiple imputation makes use of all available data and reduces potential bias associated with missing values under the missing‐at‐random assumption, the results from the imputed dataset were considered the primary findings, whereas the complete‐case analysis provided supportive evidence of their robustness (Supporting Table 2).

**Model fitness and comparison:** Model fitness statistics indicated that including both individual‐ and community‐level variables substantially improved model fit compared to Model I (null model). The log‐likelihood ratio (LLR) and deviance (−2LLR) showed that Model II (individual level) and Model IV (full model) had much higher LLR and lower deviance than the null model, whereas Model III (community level) showed only modest improvement. Among all models, the full model (Model IV) had the lowest deviance (−2LLR = 149,497.92), as well as the lowest AIC (AIC = 149,557.9) and BIC (BIC = 149,843.3), indicating the best overall fit and optimal balance between model fit and complexity. These results suggest that individual‐level variables drive much of the improvement in fit, while incorporating community‐level variables further refines the model by capturing cluster‐level variation, making Model IV the preferred model for inference. Consistent with this, the likelihood ratio test comparing the multilevel Poisson regression model with a single‐level model showed a statistically significant improvement in fit after including a random intercept at the cluster level (*χ* ^2^ (1) ≈ 200, *p* < 0.001). This confirms meaningful between‐cluster variation in the outcome and supports the inclusion of community‐level factors in the model. The estimated cluster‐level variance ($\sigma_{u}^{2}$ = 0.0072; 95% CI: 0.0051–0.0102) was significantly different from zero, further validating the use of a multilevel modeling approach to account for clustering in child deworming uptake (Table 3).

**TABLE 3:** Factors associated with deworming among children aged 12–59 months.

Variable		Model I (null model)	Model II (individual‐level factors)	Model III (community‐level factors)	Model IV (individual‐ and community‐level factors)
Individual‐level factors	Categories		APR (95% CI)	APR (95% CI)	APR (95% CI)
Women’s age (year)	15–24		1		1
	25–34		1.04 (1.02–1.06)		**1.04 (1.02–1.06)**
	35–49		1.08 (1.06–1.11)		**1.08 (1.06–1.1)**

Marital status	Married		1		1
	Unmarried		1.05 (1.03–1.07)		**1.04 (1.02–1.06)**

Children’s age (months)	12–35		1		1
	36–59		1.09 (1.08–1.11)		**1.09 (1.08–1.11)**

Children’s sex	Males		1		1
	Females		1.0 (0.99–1.02)		1.0 (0.99–1.02)

Women’s education	No education		1		1
	Primary		1.2 (1.17–1.24)		**1.19 (1.16–1.22)**
	Secondary		1.23 (1.19–1.3)		**1.2 (1.18–1.26)**
	Higher		1.31 (1.26–1.36)		**1.29 (1.2–1.34)**

Women’s literacy status	Literate		1		1
	Illiterate		0.92 (0.9–0.93)		**0.93 (0.91–0.95)**

Household size	1–4		1		1
	≥ 5		0.96 (0.95–0.98)		**0.97 (0.95–0.98)**

Household head	Males		1		1
	Females		0.98 (0.96–1.0)		0.98 (0.97–1.0)

Women’s decision‐making on health	Jointly/others		1		1
	Own		1.07 (1.06–1.09)		**1.07 (1.05–1.09)**

Wealth index	Poor		1		1
	Middle		1.07 (1.05–1.1)		**1.07 (1.04–1.09)**
	Rich		1.14 (1.1–1.17)		**1.13 (1.1–1.16)**

Media exposure	No		1		1
	Yes		1.07 (1.05–1.09)		**1.06 (1.04–1.08)**

Water source	Improved		1		1
	Unimproved		1.05 (1.02–1.07)		**1.05 (1.03–1.08)**

Sanitation facility	Improved		1		1
	Unimproved		0.99 (0.97–1.0)		0.99 (0.97–1.01)

Birth order	First		1		1
	Subsequent		1.01 (0.99–1.03)		1.01 (0.99–1.03)

ANC visits	No ANC		1		1
	1–2 visits		1.1 (1.07–1.2)		**1.1 (1.06–1.16)**
	3 visits		1.2 (1.15–1.25)		**1.19 (1.15–1.24)**
	4 and above		1.27 (1.2–1.32)		**1.26 (1.2–1.31)**

Vitamin A supplementation	No		1		1
	Yes		2.76 (2.68–2.84)		**2.75 (2.67–2.83)**

Presence of diarrhea	No		1		1
	Yes		1.06 (1.04–1.09)		**1.07 (1.05–1.09)**

*Community-level factors*					
Community literacy	Low			1	1
	High			1.27 (1.2–1.32)	**1.09 (1.06–1.12)**

Community media exposure	Low			1	1
	High			1.12 (1.08–1.2)	**1.04 (1.02–1.07)**

Community wealth index	Low			1	1
	High			0.97 (0.94–1.0)	0.99 (0.96–1.01)

Residence	Urban			1	1
	Rural			0.84 (0.81–0.86)	1.01 (0.98–1.03)

Distance to health facility	Not a big problem			1	1
	Big problem			0.83 (0.81–0.85)	**0.94 (0.92–0.96)**

*Model fitness statistics*					
Log‐likelihood ratio (LLR)		−82328.58	−74806.66	−81735.27	−74748.96
Device (−2LLR)		164,657.16	149,613.32	163,470.54	149,497.92
AIC		164,661.2	149,663.3	163,484.5	149,557.9
BIC		164,680.2	149,901.1	163,551.2	149,843.3

*Random effect results*					
Cluster‐level variance ($\sigma_{u}^{2}$)		0.057422	0.0088462	0.0327908	0.0071869
ICC		1.7%	0.27%	0.99%	0.22%
MRR		1.257	1.094	1.188	1.084
PCV		—	84.6%	42.9%	87.5%

*Comparison between single-level Poisson model and multilevel Poisson model*					
Model		Cluster‐level variance ($\sigma_{u}^{2}$)	Log‐likelihood/pseudo‐log‐likelihood	Likelihood ratio *χ* ^2^ (1)	*p* value
Single‐level Poisson		—	−27099.8 (pseudo)	—	—
Multilevel Poisson		0.00719 (95% CI: 0.00509–0.01015)	—	≈ 200	< 0.001

*Note:* Bold values indicate statistically significant associations at *p* < 0.05.

**Measures of variation results:** The measures of variation indicate meaningful between‐cluster differences in child deworming. In the null model, the cluster‐level variance ($\sigma_{u-\text{null}}^{2}$) was 0.057422, with ICC of 1.7% and MRR of 1.26, suggesting that children within the same cluster were slightly more similar in deworming status than those from different clusters. After including individual‐level factors, the cluster variance decreased to 0.0088462 (ICC = 0.27%, MRR = 1.09), accounting for approximately 84.6% of the between‐cluster variance. When only community‐level factors were included, the variance dropped to 0.0327908 (ICC = 0.99%, MRR = 1.19), explaining 42.9% of the cluster‐level variation. In the full model, which incorporated both individual‐ and community‐level variables, the residual cluster variance was minimal ($\sigma_{u-\text{model}}^{2}$ = 0.0071869; ICC = 0.22%; MRR = 1.08), indicating that 87.5% of the between‐cluster variance was explained. A small residual heterogeneity remained, confirming the appropriateness of a random‐intercept multilevel Poisson regression model to account for clustering. Furthermore, a likelihood ratio test demonstrated that including random intercepts significantly improved model fit (*χ* ^2^ (1) ≈ 200, *p* < 0.001), supporting the use of multilevel Poisson regression to accurately estimate individual‐ and community‐level effects in the hierarchical DHS data (Table 3).

### 3.6. Factors Associated With Deworming Among Children Aged 12–59 Months

Prior to conducting multilevel Poisson regression, multicollinearity among the independent variables was assessed. The results indicated low multicollinearity, with all VIFs below 5. The highest VIFs were observed for maternal education (1.94), literacy status (1.88), and wealth index (1.84), while the remaining variables ranged from 1.00 to 1.51 (mean VIF = 1.30), suggesting that the independent variables were not highly correlated and suitable for inclusion in the regression model.

In the full model (Model IV), which accounted for both individual‐ and community‐level characteristics, several factors were significantly associated with deworming among children aged 12–59 months. Children of women aged 25–34 years (APR = 1.04, 95% CI: 1.02–1.06) and 35–49 years (APR = 1.08, 95% CI: 1.06–1.10) were more likely to be dewormed than those whose mothers were aged 15–24 years. Children of unmarried women were slightly more likely to receive deworming (APR = 1.04, 95% CI: 1.02–1.06) than those of married women. Older children (36–59 months) had a higher prevalence of deworming (APR = 1.09, 95% CI: 1.08–1.11) than younger children (12–35 months). Compared to mothers with no education, those with primary (APR = 1.19, 95% CI: 1.16–1.22), secondary (APR = 1.20, 95% CI: 1.18–1.26), and higher education (APR = 1.29, 95% CI: 1.20–1.34) were more likely to have dewormed their children. Illiterate mothers were less likely to deworm their children than literate ones (APR = 0.93, 95% CI: 0.91–0.95). Children from larger households (≥ 5 members) had slightly lower prevalence of receiving deworming (APR = 0.97, 95% CI: 0.95–0.98). Women who independently made decisions regarding their own health care were 7% more likely to deworm their children than those whose healthcare decisions were made jointly with their husbands or by others (APR = 1.07, 95% CI: 1.05–1.09). Similarly, children from middle‐income households (APR = 1.07, 95% CI: 1.04–1.09) were 7% more likely, and those from rich households (APR = 1.13, 95% CI: 1.10–1.16) were 13% more likely, to have been dewormed compared to children from poor households. Exposure to media was also associated with a higher likelihood of deworming (APR = 1.06, 95% CI: 1.04–1.08) compared to those who were not exposed. Interestingly, children from households using unimproved water sources had a slightly higher likelihood of being dewormed (APR = 1.05, 95% CI: 1.03–1.08), whereas sanitation facility type and birth order were not significantly associated. The likelihood of a child being dewormed increased with the number of ANC visits; specifically, children whose mothers attended four or more ANC visits were 26% more likely to receive deworming medication (APR = 1.26, 95% CI: 1.20–1.31) compared to those whose mothers had no ANC visits. Children who received vitamin A supplementation were almost three times more likely to be dewormed (APR = 2.75, 95% CI: 2.67–2.83). Additionally, children who had diarrhea were more likely to receive deworming (APR = 1.07, 95% CI: 1.05–1.09). At the community level, residing in areas with high literacy (APR = 1.09, 95% CI: 1.06–1.12) and high media exposure (APR = 1.04, 95% CI: 1.02–1.07) was significantly associated with a higher likelihood of deworming, whereas mothers who perceived distance to a health facility as a major problem were less likely to deworm their children (APR = 0.94, 95% CI: 0.92–0.96) (Table 3).

## 4. Discussion

This study examined the coverage and determinants of deworming among children aged 12–59 months in sub‐Saharan Africa using recent DHS data collected between 2021 and 2024. The overall deworming coverage was 48.98%, indicating that nearly half of eligible children had received deworming medication. Despite global and regional commitments to control STH infections, this finding showed that coverage remains below the WHO target of at least 75% preventive chemotherapy coverage among at‐risk populations [19, 20]. According to the WHO guidelines, preschool‐aged children should receive deworming treatment once a year when community prevalence exceeds 20% and twice a year when it exceeds 50% [6]. However, the present findings indicate that, despite these recommendations, coverage across sub‐Saharan Africa remains suboptimal and unevenly distributed. This gap between guideline recommendations and actual coverage underscores persistent challenges in program implementation, accessibility, and the equitable delivery of deworming interventions across the region [13–15].

Significant inter‐country variations were observed in this study, with coverage ranging from 32.0% in Burkina Faso to 66.0% in Kenya. Countries such as Kenya, Lesotho, DRC, and Madagascar reported relatively high coverage levels above 50%, reflecting stronger programmatic efforts and sustained support from international partners. These countries may benefit from coordinated campaigns involving WHO, UNICEF, and other implementing partners that integrate deworming with broader child health services, such as vitamin A supplementation and immunization, as well as WASH interventions [5, 6, 13–15]. Such integration reduces reinfection rates, enhances program sustainability, and improves overall effectiveness. Evidence from UNICEF‐supported campaigns, including those in Ethiopia, indicates that combining deworming with other child health services substantially increases coverage and cost‐effectiveness [14, 15]. In contrast, countries such as Burkina Faso, Senegal, and Mozambique reported the lowest coverage, which may be linked to health system constraints, limited access to maternal and child health services, resource shortages, and inadequate community mobilization [9–12]. Expanding the integration of deworming with broader child health– and context‐specific approaches, particularly in low‐performing countries, is essential to accelerating progress toward universal deworming coverage across sub‐Saharan Africa.

The overall coverage in this study is slightly higher than that reported in previous research conducted before 2020, which found deworming coverage of 45% among children under five in sub‐Saharan Africa, with country‐specific estimates ranging from 41.8% in Malawi to 50.5% in Lesotho [9]. This increase may partly reflect the more refined and accurate estimates provided by the present analysis, which adheres to the revised DHS guideline introduced after 2020, limiting the deworming indicator to children aged 12–59 months [16]. By focusing on the eligible age group, the current findings offer a more precise reflection of deworming coverage across sub‐Saharan Africa. Programmatic factors, such as variations in the integration of deworming with other health services, may contribute to these disparities in coverage [13–15]. The overall deworming coverage in this study is lower than that reported in a previous multicountry DHS analysis among children aged 12 to 59 months, which estimated coverage at 54.1% in East Africa [10]. This lower coverage is likely due to interrelated contextual, programmatic, and sociodemographic factors. Contextual challenges, such as conflict, displacement, and residence in remote areas, can disrupt service delivery and limit access to campaigns. Behavioral and socioeconomic factors, including caregiver education, awareness, and knowledge of deworming programs, as well as poverty and limited media exposure, further reduce uptake [9–12]. Programmatic factors, such as variations in national program design and campaign frequency [5, 6], also contribute to regional disparities in coverage.

Consistent with prior studies [9–12], children of mothers with higher education levels were substantially more likely to receive deworming. Educated mothers are more likely to be knowledgeable about preventive health measures, access health services, and adhere to recommended child health practices. Similarly, children of literate mothers who made independent healthcare decisions were significantly more likely to be dewormed, highlighting the critical role of women’s empowerment and education in improving child health outcomes. Maternal age was also positively associated with childhood deworming, as children with older mothers had a higher prevalence of receiving deworming medication than children with younger mothers. This finding was consistent with previous studies, which reported that older mothers were more likely to have their children receive deworming medication than younger mothers [9–12]. This may be attributed to greater parenting experience, improved knowledge of child health practices, and increased familiarity with preventive health services among older women. Children of unmarried women showed a slightly higher likelihood of receiving deworming than those of married women. Similarly, older children (36–59 months) were more likely to receive deworming than younger children (12–35 months), possibly because they have greater exposure to community‐based deworming campaigns, routine child health services, and repeated opportunities for contact with healthcare providers. Children from middle‐ and high‐income households and those exposed to mass media also had higher deworming rates, reflecting the importance of economic resources and access to information in promoting preventive health behaviors [9–12]. At the community level, higher literacy and media exposure further enhanced deworming uptake, suggesting that broader social environments shape awareness and acceptance of health interventions. Children whose mothers attended at least one ANC visit were more likely to be dewormed, consistent with evidence that ANC visits provide opportunities for health education, counseling, and linkage to preventive child health services [9–12]. Children who received vitamin A supplementation were almost three times more likely to be dewormed, representing the strongest association observed in this study. This finding strongly suggests that deworming is frequently delivered through integrated child health platforms, such as Child Health Days and combined campaigns that provide vitamin A supplementation, immunization, and other preventive services simultaneously. The result highlights the success of service integration as a strategy for increasing deworming coverage and suggests that expanding integrated delivery platforms could be one of the most effective approaches for accelerating progress toward WHO coverage targets in sub‐Saharan Africa [13–15]. The positive association between recent diarrhea and deworming should be interpreted cautiously because diarrhea in DHS is measured for the 2 weeks preceding the survey, whereas deworming refers to the previous 6 months. Therefore, the finding does not establish a temporal relationship. More plausibly, diarrhea may act as a proxy for healthcare‐seeking behavior [21], whereby caregivers who bring children to health facilities for diarrhea treatment are also more likely to access or receive deworming services [5, 6, 13]. Children from larger households and those whose mothers reported distance to a health facility as a major barrier were less likely to be dewormed. These findings agree with previous research showing that household crowding and limited physical access to health services reduce preventive care use [9–12]. Targeted outreach programs and community‐based service delivery could help address these barriers.

Interestingly, children from households using unimproved water sources had a slightly higher likelihood of being dewormed. This finding is counterintuitive and should be interpreted cautiously. One possible explanation is that deworming activities may be preferentially implemented in communities perceived to be at higher risk of STHI; however, the present study did not include programmatic data to confirm such targeting. Therefore, this interpretation should be considered a hypothesis rather than evidence of targeted implementation.

### 4.1. Strengths and Limitations of the Study

This study has several notable strengths and limitations that should be considered when interpreting the findings. A key strength is the use of recent and standardized DHS data collected between 2021 and 2024. The DHS employs rigorous sampling and data collection procedures, ensuring nationally representative and comparable datasets across countries and time periods. The inclusion of a large sample of children aged 12 to 59 months enhances both the statistical power and external validity of the results, allowing meaningful regional generalizations. In addition, by applying the revised DHS‐8 definition of deworming, which focuses on children aged 12 to 59 months, the study aligns with updated international standards and produces findings that are more relevant to current policy frameworks. Methodologically, the use of Poisson regression with a log link and robust variance is a strength, as it provides more appropriate and interpretable estimates of prevalence ratios for common outcomes than logistic regression. The identification and imputation of missing values helped minimize the potential bias associated with incomplete data and improved the efficiency and reliability of the estimates. To assess the impact of this approach, a sensitivity analysis comparing the complete‐case and multiply imputed multilevel Poisson regression models was conducted. The results were highly consistent, with most APRs showing similar directions, magnitudes, and statistical significance across both analyses, indicating that the use of multiple imputation did not materially alter the study conclusions. Despite these strengths, the study also has certain limitations. The cross‐sectional nature of the DHS data limits the ability to infer causal relationships between explanatory factors and deworming coverage, as temporal sequences cannot be established. Moreover, only 11 sub‐Saharan African countries were included, which may affect the generalizability of the findings to the entire region. Deworming information was obtained through maternal self‐reports, which may be subject to recall or social desirability bias, potentially leading to overestimation or underestimation of the true coverage levels. Although multiple observed covariates were controlled for, residual confounding from unmeasured factors such as local program quality, community health interventions, or caregiver awareness cannot be ruled out. Although multiple imputation was used to address missing data and sensitivity analyses were performed, the validity of the imputed estimates depends on the assumption that the data were missing at random. As the ANC variable had substantial missingness (56.0%), bias may persist if this assumption was not met. Additionally, the pooled multicountry analysis, though valuable for regional insights, may obscure important contextual variations in health system capacity and program implementation across individual countries. As a secondary data analysis, the study was also limited by the variables available in the DHS dataset, excluding programmatic and contextual indicators such as drug supply consistency, deworming campaign frequency, and health service accessibility.

## 5. Conclusion

Nearly half of eligible children in sub‐Saharan Africa received deworming medication, with substantial variation across countries. Children of educated and empowered mothers, those from wealthier households, and those whose mothers attended ANC or received vitamin A supplementation were more likely to be dewormed. Programmatically, the findings suggest that integrating deworming with existing maternal and child health services, strengthening health education through media, and improving access in poor and remote communities are critical for increasing coverage. Enhancing women’s education and decision‐making capacity, alongside targeted community outreach, can help sustain progress toward controlling helminth infections in the region.

**Nomenclature**
DHS: Demographic and Health Survey
UNICEF: United Nations International Children’s Emergency Fund
WHO: World Health Organization
STH: Soil‐transmitted helminth
WASH: Water, sanitation, and hygiene
ANC: Antenatal care
ICC: Intraclass correlation coefficient
MRR: Median rate ratio
PCV: Proportional change in variance
AIC: Akaike information criterion
BIC: Bayesian information criterion
DRC: Democratic Republic of the Congo
APR: Adjusted prevalence ratio
CI: Confidence interval
Fig: Figure
VIF: Variance inflation factor.

## Author Contributions

Temesgen Gebeyehu Wondmeneh conceived the idea, extracted the data, performed the data analysis, and drafted the manuscript. He interpreted the analysis and revised the manuscript.

## Funding

The author did not receive any funding.

## Disclosure

The author has read and approved the final version.

## Ethics Statement

Permission to use the data for this analysis was obtained from the DHS Program. Access to de‐identified, anonymized datasets was granted through a formal request (https://dhsprogram.com/data/Access-Instructions.cfm). Data were securely stored and analyzed in aggregate, following DHS data use policies and international ethical standards.

## Consent

The DHS Program obtained informed consent from all participants during data collection.

## Conflicts of Interest

The author declares no conflicts of interest.

## Supporting Information

Additional supporting information can be found online in the Supporting Information section.

## Supporting information


**Supporting Information 1** Supporting Table 1 shows the summary and pattern of missing data for missing variables included in the analysis.

**Supporting Information 2** Supporting Table 2 shows the complete case analysis.

**Supporting Information 3** Supporting information 1 shows STROBE guideline for cross‐sectional studies.

## Data Availability

The data used in this study were obtained from the DHS Program for the survey years 2021–2024. The datasets are publicly available upon reasonable request and approval from the DHS Program. Researchers can apply for access through the DHS website at https://dhsprogram.com/data/dataset_admin/index.cfm.
