# Supplementary material for: Deworming Coverage and Associated Factors Among Children Aged 12–59 Months in Sub‐Saharan Africa: A Multilevel Poisson Regression Analysis Using Demographic and Health Survey Data, 2021–2024
Source: J Trop Med. 2026 Sep 22;2026:7289470. doi: 10.1155/jotm/7289470 (PMC13598377; doi:10.1155/jotm/7289470)
Supplement: Supplementary file 1 [file JOTM-2026-7289470-s001.docx]

### Supplementary Table 1. Summary and pattern of missing data across variables

| Variable | Missing (Obs=.) | Observed (Obs>.) | % Missing | Type | Common Missing Pattern (%) |
| --- | --- | --- | --- | --- | --- |
| Decision-making power | 12,641 | 88,358 | 12.5% | Binary | Often with ANC (5–6%) |
| Diarrhea | 459 | 100,540 | 0.5% | Binary | Rare (<1%) |
| Vitamin A supplementation | 612 | 100,387 | 0.6% | Binary | Occasional with ANC (6%) |
| Antenatal care visits (ANC) | 56,582 | 44,417 | 56.0% | Categorical | Mainly missing alone (46%) |
| Distance to health facility | 6,974 | 94,025 | 6.9% | Binary | Occasional with ANC (3–4%) |
