# Supplementary material for: Deworming Coverage and Associated Factors Among Children Aged 12–59 Months in Sub‐Saharan Africa: A Multilevel Poisson Regression Analysis Using Demographic and Health Survey Data, 2021–2024
Source: J Trop Med. 2026 Sep 22;2026:7289470. doi: 10.1155/jotm/7289470 (PMC13598377; doi:10.1155/jotm/7289470)
Supplement: Supplementary file 2 [file JOTM-2026-7289470-s002.docx]

| Supplementary Table 2 Complete case analysis results for the final model of individual- and community-level factors | | |
| --- | --- | --- |
| Variable | | Model-IV (Individual and community-level factors) |
| **Individual-level factors** | Categories | APR (95%CI) |
| Women’s age (year) | 15-24 | 1 |
|  | 25-34 | 1.03 (0.998–1.06) |
|  | 35-49 | **1.07 (1.03–1.10)** |
| Marital status | Married | 1 |
|  | Unmarried | **1.03 (1.02-1.5)** |
| Children’s age ( months) | 12-35 | 1 |
|  | 36-59 | **1.39 (1.34–1.44)** |
| Children’s sex | Males | 1 |
|  | Females | 0.99 (0.97-1.01) |
| Women’s education | No education | 1 |
|  | Primary | **1.16 (1.12–1.20)** |
|  | Secondary | **1.19 (1.14–1.24)** |
|  | Higher | **1.18 (1.11–1.25)** |
| Women’s literacy status | Literate | 1 |
|  | Illiterate | 0.94 (0.91–0.96) |
| Household size | 1-4 | 1 |
|  | ≥ 5 | 0.99 (0.97-1.01) |
| Household head | Males | 1 |
|  | Females | 0.99 (0.96-1.02) |
| **Women’s decision-making on health** | Jointly/Others | 1 |
|  | Own | **1.06 (1.03–1.09)** |
| Wealth index | Poor | 1 |
|  | Middle | **1.08 (1.04–1.11)** |
|  | Rich | **1.16 (1.12–1.20)** |
| Media exposure | No | 1 |
|  | Yes | **1.03 (1.01–1.06)** |
| Water source | Improved | 1 |
|  | Unimproved | **1.07 (1.04–1.10)** |
| Sanitation facility | Improved | 1 |
|  | Unimproved | 1.0 (0.97-1.02) |
| Birth order | First | 1 |
|  | Subsequent | 1.0 (0.97-1.03) |
| ANC visits | No ANC | 1 |
|  | 1-2 visits | **1.20 (1.13–1.28)** |
|  | 3 visits | **1.29 (1.22–1.37)** |
|  | 4 and above | **1.24 (1.18–1.31)** |
| Vitamin A supplement | No | 1 |
|  | Yes | **2.54 (2.44–2.63)** |
| Presence of diarrhea | No | 1 |
|  | Yes | **1.05 (1.02–1.08)** |
| **Community level factors** | |  |
| Community literacy | Low | 1 |
|  | High | **1.06 (1.02–1.09)** |
| Community media exposure | Low | 1 |
|  | High | 1.03 (1.0-1.06) |
| Community wealth index | Low | 1 |
|  | High | 1.0 (0.96-1.03) |
| Residence | Urban | 1 |
|  | Rural | 0.99 (0.95-1.02) |
| Distance to health facility | Not big problem | 1 |
|  | Big problem | **0.96 (0.93–0.98)** |

Note: Bold values indicate statistically significant associations at p < 0.05.
